# An audit of secondary prevention for peripheral arterial disease in primary care — scope for improved collaboration between vascular surgery and general practitioners

**DOI:** 10.1007/s11845-023-03362-1

**Published:** 2023-04-26

**Authors:** Megan Power Foley, Muhammad Tubassam, Stewart R. Walsh

**Affiliations:** 1 Department of Vascular Surgery, University College Hospital Galway, Newcastle Road, Dublin 8, Dublin, H91YR71 Ireland; 2 School of Medicine, University of Galway, Galway, Ireland; 3 National Surgical Research Support Centre, Royal College of Surgeons in Ireland, Dublin, Ireland

**Keywords:** Intermittent claudication, Medical management, Peripheral arterial disease, Primary care, Risk factor modification, Secondary prevention

## Abstract

**Background:**

Symptomatic peripheral arterial disease (PAD) is a common cause for referral from primary care to vascular surgery. Best medical therapy (BMT), encompassing anti-platelets, statins, smoking cessation, blood pressure and glycaemic control, is a cornerstone of PAD management. However, these easily modifiable risk factors are often left unaddressed between referral and clinic review.

**Methods:**

A prospective audit of electronic ‘Healthlink’ referrals by GPs to the vascular department for symptomatic PAD between July 2021 and June 2022 was performed. Referrals were individually reviewed for demographics, symptoms, medical history, smoking status and medications. An information leaflet on BMT was posted to all GP practices in the Soalta region as part of an educational intervention, with plans to re-audit after 6 months.

**Results:**

One-hundred-and-seventy referrals were analysed. The median age was 68.5 years (range 33–94) and 69% (*n* = 117) were male. The typical vasculopath comorbidity profile was noted. Fifty-two percent (*n* = 88) were referred with claudication-type pain and 25% (*n* = 43) with critical limb ischaemia (CLI). Twenty-eight percent (*n* = 33) were active smokers and 31% (*n* = 36) had no smoking status documented. Regarding BMT, only 34.5% (*n* = 40) and 52% (*n* = 60) were on anti-platelets and statins, respectively. Suspected CLI was not significantly associated with BMT prescription at referral (*p* = 0.664). Only eleven referral letters mentioned risk factor optimisation.

**Conclusions:**

Our first-cycle results identified significant scope for improvement in community-based risk factor modification for PAD referrals. We aim to continue supporting and educating our colleagues that effective medical management can start safely in primary care and further explore the barriers preventing this.

## Introduction

Peripheral arterial disease (PAD) is a systemic atherosclerotic process affecting up to 20% of western adults older than 50 years [1]. It shares common risk factors and pathophysiology with coronary and cerebrovascular disease, and these conditions often co-exist, creating a uniquely high-risk patient cohort with high rates of cardiovascular morbidity. Advanced age, hypertension, dyslipidaemia, diabetes mellitus, and cigarette smoking are all associated with PAD. Notably, symptomatic PAD is associated with a 10–25% 5-year all-cause mortality rate [1, 2].

While the presentation of PAD ranges from asymptomatic to intermittent claudication to chronic limb-threatening ischaemia (CLTI), best medical therapy remains a cornerstone of management across all disease stages. Encompassing anti-platelet monotherapy and statins, optimising blood pressure and glycaemic control, and promoting smoking cessation and exercise to stimulate collateral development, medical management of PAD is evidence-based, safe and effective [3–5]. However, despite explicit guidelines from multiple international cardiovascular expert groups outlining the benefits of medical treatment for PAD, optimisation of risk factors in the primary care setting remains poor [6–8].

## Methods

We performed a prospective audit of new GP referrals for suspected PAD to the vascular surgery department. A random selection of referral letters received between July 2021 and June 2022 were individually reviewed for demographic characteristics, reported symptoms, medical history, smoking status and medications. Referrals were included in the analysis if the described symptoms were consistent with PAD, as determined by a vascular specialist registrar, or if the referral specifically included the phrases “claudication”, “ischaemia”, “PAD” or “arterial ulcer”. Attention to modifiable risk factors by GPs was noted, namely smoking cessation and the commencement of best medical therapy.

Data were anonymised and entered into a password protected worksheet. Statistical analysis was performed using Statistical Package for the Social Sciences (SPSS) software Version 27.0 (IBM SPSS Inc., IBM Corp., Armonk, NY, USA). Normally distributed continuous data were expressed as mean ± standard deviation (SD), while median (range) was used to describe the abnormally distributed continuous data. Categorical variables were presented as count and percent. Chi-square tests and Odds Ratio were used to analyse categorical variables. A *p*-value of < 0.05 was considered to be statistically significant.

## Results

In total, 170 referrals were included in the analysis. The median age was 68.5 years (range 33–94) and 69% (*n* = 117) were male. The referral indication was claudication-type pain in 61% (*n* = 104) and CLTI in 25% (*n* = 43) with the remainder reporting non-specific symptoms (e.g. cold feet, blue discoloration, paraesthesia, asymptomatic but pulses absent). Notably, 23.5% (*n* = 40) of referrals did not report vascular examination findings and peripheral pulse status. The typical vasculopath comorbidity profile was present (Table 1).

**Table 1:** Count and percentage of comorbidities, symptoms, examination findings, smoking status and active medications documented in GP referral letters

	**n**	**%**
**Comorbidities**		
Hypertension IHD Diabetes Dyslipidaemia COPD Stroke CKD	113 73 59 54 15 11 9	66.5 43 35 32 9 6.5 5
**Reported symptoms**		
Claudication Rest Pain Tissue Loss Miscellaneous	104 35 17 39	61 20.5 10 23
**Vascular examination**		
Pulses Present Pulses Absent Not Reported	61 69 40	36 40.5 23.5
**Smoking status**		
Active Smoker Ex-Smoker Never Smoker Not Reported	55 30 35 50	32.5 17.5 20.5 29.5
**Medications**		
Anti-Platelets Statins Anti-Coagulation	62 88 28	36.5 52 16.5

Fifty-five referrals (32.5%) reported patients were active smokers, though only nineteen referenced discussing smoking cessation and eight GPs prescribed nicotine-replacement therapy. Notably, fifty referrals (29.5%) did not mention smoking status at all. Compared to non-smokers, active smokers were significantly younger at the time of referral (median 63.0 vs 75.0 years, *p* < 0.001) and were more likely to present with claudication-type pain (69% vs 49%, *p* = 0.028).

Regarding medical management, only 35% (*n* = 60) of referral were prescribed optimal “Best Medical Therapy”. Suspected CLTI was not associated with BMT prescription at referral (*p* = 0.664). However, concurrent IHD was significantly associated with BMT, and patients with known IHD were significantly more likely to be prescribed secondary prevention (*p* < 0.001, OR 7.681 95% CI 3.768–15.658). Seventeen GPs commenced vasoactive medications, like Cilostazol or Pentoxifylline, at the time of consultation — however, nine of these patients were not even on best medical therapy.

## Discussion

### Risk factor optimisation is critical to PAD management

Our initial audit cycle results demonstrated that there is significant scope for improvement in early risk factor optimisation for patients with suspected PAD. While the vascular surgery service plays a major role in delivering specialist care, secondary prevention should be commenced immediately in the community once PAD is suspected. The evidence supporting low-dose antiplatelet monotherapy (75 mg Aspirin or Clopidogrel), lipid-lowering therapy with statins, optimising blood pressure and glycaemic control, and smoking cessation for treatment of PAD is of the highest quality possible [3–5]. In particular, smoking is associated with a two- to threefold increased risk of PAD that persists for up to 30 years post-cessation; as such, every patient encounter should be treated as opportunity for cessation counselling [1].

The natural history of PAD suggests that approximately 20% of claudicants will progress to CLTI and ultimately up to 25% will require extremity amputation [9]. Notably, recent studies have reported that revascularisation attempts for claudication does not durably improve quality of life and is associated with higher risk of progression to CLTI and limb loss [10–12]. As such, emphasising the importance of early medical management to all stakeholders involved in PAD care is critical. In addition to combating PAD progression, medical optimisation in this high-risk cohort significantly reduces the rates of cardiovascular and limb events over the long term, thereby reducing PAD-related morbidity, mortality and healthcare costs [7, 13, 14].

### Poor uptake of secondary prevention

In our cohort, only 35% of patients were prescribed BMT at the time of vascular referral. Notably, patients with previously diagnosed IHD were seven times more likely to be receiving BMT — likely because it has already been prescribed by a cardiologist (*p* < 0.001, OR 7.681 95% CI 3.768–15.658). Poor uptake of secondary prevention measures in primary care is not unique to Ireland, with studies from Northern Ireland, New Zealand and the Netherlands all reporting underutilisation of medical management for PAD [15–17]. A multi-centre study of 656 new referrals to vascular surgery units across the UK reported 61.7% and 61.5% of patients were prescribed anti-platelets and statins at the time of referral, respectively [18].

Concerningly, there also is a marked lack of awareness of PAD and its sequalae amongst the Irish patient population, creating a significant challenge for general practitioners and vascular surgeons [19, 20]. A large survey of Irish diabetics demonstrated that only 12–16% were aware that peripheral arterial disease and major amputation were common complications of their condition. Similarly, less than half those surveyed believed that lifestyle modification could reduce their cardiovascular risks [21]. A collaborative partnership between primary and tertiary care is essential to successfully manage these high-risk, low-awareness patients, particularly in the context of unacceptably long waiting lists for outpatient appointments. While patients with suspected CLTI are triaged and seen expediently, claudicants inevitably experience protracted delays before clinic review — time that should be spent optimising their vascular risk factors.

### Building a PAD partnership between primary and tertiary care

As the majority of PAD is suitable for community-based care, with vascular surgery becoming involved in the case of CLTI, GPs must take a more active role in secondary prevention efforts. To enhance vascular care, we posted a brief PAD risk factor optimisation information sheet to all GP practices within the Saolta group, highlighting the role of medical management for PAD. We suspect that an element of GP colleagues’ reluctance to start BMT is a conscientious desire to have their suspected diagnosis confirmed by a vascular specialist. However, it is in the patients’ best interest to start secondary prevention without delay. The re-audit will take place after a 6-month wash-out period to gauge the impact of this intervention.
